# Polymorphisms of XRCC1 genes and risk of nasopharyngeal carcinoma in the Cantonese population

**DOI:** 10.1186/1471-2407-6-167

**Published:** 2006-06-26

**Authors:** Yun Cao, Xiao-Ping Miao, Ma-Yan Huang, Ling Deng, Li-Fu Hu, Ingemar Ernberg, Yi-Xin Zeng, Dong-Xin Lin, Jian-Yong Shao

**Affiliations:** 1 The State Key Laboratory of Cancer Biology of Southern China and Department of Experiment, Sun Yat-Sen University Cancer Center, Guangzhou 510060, China; 2 Department of Etiology and Carcinogenesis, Cancer Institute, Chinese Academy of Medical Sciences and Peking Union Medical College, Beijing 100021, China; 3 Microbiology and Tumor Biology Center, Karolinska Institute, Stockholm S171 77, Sweden

## Abstract

**Background:**

Nasopharyngeal carcinoma (NPC) is one of the most common cancers in southern China. In addition to environmental factors such as Epstein-Barr virus infection and diet, genetic susceptibility has been reported to play a key role in the development of this disease. The x-ray repair cross-complementing group 1 (XRCC1) gene is important in DNA base excision repair. We hypothesized that two common single nucleotide polymorphisms of XRCC1 (codons 194 Arg→Trp and 399 Arg→Gln) are related to the risk of NPC and interact with tobacco smoking.

**Methods:**

We sought to determine whether these genetic variants of the XRCC1 gene were associated with the risk of NPC among the Cantonese population in a hospital-based case control study using polymerase chain reaction-restriction fragment length polymorphism analysis. We conducted this study in 462 NPC patients and 511 healthy controls.

**Results:**

After adjustment for sex and age, we found a reduced risk of developing NPC in individuals with the Trp194Trp genotype (OR = 0.48; 95% CI, 0.27–0.86) and the Arg194Trp genotype (OR = 0.79; 95% CI, 0.60–1.05) compared with those with the Arg194Arg genotype. Compared with those with the Arg399Arg genotype, the risk for NPC was not significantly different in individuals with the Arg399Gln genotype (OR = 0.82; 95% CI, 0.62–1.08) and the Gln399Gln genotype (OR = 1.20; 95% CI, 0.69–2.06). Further analyses stratified by gender and smoking status revealed a significantly reduced risk of NPC among males (OR = 0.32; 95% CI, 0.14–0.70) and smokers (OR = 0.34; 95% CI, 0.14–0.82) carrying the XRCC1 194Trp/Trp genotype compared with those carrying the Arg/Arg genotype. No association was observed between Arg399Gln variant genotypes and the risk of NPC combined with smoking and gender.

**Conclusion:**

Our findings suggest that the XRCC1 Trp194Trp variant genotype is associated with a reduced risk of developing NPC in Cantonese population, particularly in males and smokers. Larger studies are needed to confirm our findings and unravel the underlying mechanisms.

## Background

Nasopharyngeal carcinoma (NPC) is one of the most common cancers in the Cantonese population in southern China [1]. Etiological factors include Epstein-Barr virus infection, tobacco smoking, and consumption of salted fish [2-4]. In addition to environmental factors, genetic factors such as chromosomal aberrations, nucleotide polymorphisms of HLA class I and II alleles, glutathione S-transferase M1, and cytochrome P450 2E1 also play a role in the development of NPC [5-9]. Only a few people develop the disease in areas where NPC is endemic even though everyone is exposed to the same environment, suggesting that genetic differences such as single nucleotide polymorphisms (SNP) may contribute to NPC carcinogenesis.

Polymorphisms in XRCC1 have been identified at conserved sites, including two SNPs at codons 194 (Arg to Trp) and 399 (Arg to Gln) [10]. These findings suggest that since XRCC1 is essential for removing endogenous and exogenous DNA damage, the Arg399Gln substitution may result in deficient DNA repair [11,12]. On the other hand, the Trp194 allele seemed to be protective against DNA damage, although no significant difference was observed from wild-type in terms of DNA repair capacity.

Molecular epidemiological studies investigating the Arg194Trp and Arg399Gln variants of XRCC1 and their impact on cancer risk have generated inconsistent results. The Arg194 allele is associated with an increased risk of gastric cardia cancer (adjusted OR = 1.86, 95% CI, 1.09–3.20), and the Gln399 allele is associated with an increased risk of gastric cancer (adjusted OR = 1.53, 95% CI, 0.98–2.39) [13]. Olshan et. al. reported a weak increase in the risk of head and neck cancer associated with the Arg194Trp polymorphism (OR = 1.3, 95% CI, 0.6–2.9) and a decreased risk for the Arg399Gln polymorphism (OR = 0.6; CI = 0.4–1.1) [14]. Other studies have reported that these two XRCC1 variants are associated with elevated risks of esophageal cancer [15], pancreatic adenocarcinoma [16], and breast cancer [17].

In the present study, we investigated the association between the Arg194Trp and Arg399Gln variants of XRCC1, smoking status, and the risk of developing NPC in the Cantonese population living in southern China.

## Methods

### Study subjects

The study group consisted of 462 patients with histologically confirmed, untreated NPC and 511 cancer-free controls. All subjects were ethnic Cantonese living in southern China. The NPC patients were consecutively recruited from February 2000 to September 2003 at Sun Yat-Sen University Cancer Center (Canton). Disease staging was performed in accordance with the China 92 TNM staging system [18]. Population controls were selected from a community screening program for early detection of cancer. For each eligible case, we tried to match one control subject by age (± 5 years) and ethnicity (Cantonese). At recruitment, informed consent was obtained from each subject and each participant was then interviewed to solicit detailed information on demographic characteristics and lifetime history of tobacco use. Overall, 520 eligible cases and 520 eligible controls agreed to further risk factor interviews administered by a trained nurse-interviewer, with the final study consisting of 462 cases (88.8% of eligible) and 511 controls (98.3% of eligible) due to lack of information on smoking and the inability to collect blood from some subjects. Information was collected on the number of cigarettes smoked per day, the age at which the subjects started smoking, and the age at which ex-smokers stopped smoking. Pack-years smoked was calculated to indicate cumulative cigarette dose [pack-years = (cigarettes per day/20) × (years smoked)]. Smokers were considered current smokers if they had smoked up to 1 year before the date of cancer diagnosis (or up to the date of the interview for controls). This study was approved by the Hospital Review Board of Sun Yat-Sen University Cancer Center.

### XRCC1 genotyping

Genomic DNA was isolated from peripheral blood and XRCC1 genotypes at the Arg194Trp and Arg399Gln sites were determined by PCR-based restriction fragment length polymorphism as described by Xing et. al. [15]. The primers for the XRCC1 Arg194Trp polymorphism were 5' GCC AGG GCC CCT CCT TCA A 3' and 5' TAC CCT CAG ACC CAC GAG T 3', which generate a 485 bp fragment. The primers for the XRCC1 Arg399Gln polymorphism were 5'TCC TCC ACC TTG TGC TTT CT 3' and 5' AGT AGT CTG CTG GCT CTG GG 3', which generate a 517 bp fragment. PCR was performed in a 25 μl reaction mixture containing approximately 100 ng DNA, 1.0 μM each primer, 0.2 mM each dNTP, 2.0 mM MgCl_2_, 1.0 U Taq DNA polymerase with 1 × reaction buffer (Promega, Madison, WI), and 2% dimethyl sulfoxide. Amplification of the Arg194Trp site was carried out under the following conditions: an initial melting step of 2 min at 95°C, followed by 35 cycles of 30 s at 94°C, 30 s at 57°C, and 45 s at 72°C, and a final elongation of 7 min at 72°C. The reaction conditions for the Arg399Gln site were identical except that the annealing temperature was 61°C.

The restriction enzymes *PvuII *and *BcnI *(New England Biolabs, Beverly, MA) were used to distinguish the Arg194Trp and Arg399Gln polymorphisms, respectively. The wild-type Arg194Trp allele generates a single band representing the entire 485 bp fragment and the variant 194Trp allele generates 396 and 89 bp fragments. At the Arg399Gln site, the wild-type allele (Arg/Arg) generates 2 DNA bands (384 and 133 bp), the variant allele (Gln/Gln) has a single 517 bp fragment, and the heterozygote (Arg/Gln) displays all 3 bands (517, 384, and 133 bp). The restriction products were analyzed by electrophoresis on a 2% agarose gel containing ethidium bromide. To verify the results, 15% of the samples were randomly selected for duplicate analyses for 63 cases and 74 controls for the codon 194 polymorphism, and for 64 cases and 75 controls for the codon 399 polymorphism. The genotyping results were concordant with all duplicate samples.

### Statistical analysis

The Hardy-Weinberg Equilibrium was tested to compare the observed and expected genotype frequencies among cases and controls, respectively. χ^2 ^tests were used to examine the differences in genotype distributions between cases and controls. The associations between XRCC1 polymorphisms and risk of NPC were estimated by odds ratios (OR) and their 95% confidence intervals (CI), which were calculated by unconditional logistic regression. The ORs were adjusted for age, gender, and smoking status. All analyses were performed using the Statistical Analysis System (Version. 6.12, SAS Institute, Cary, NC). Bonferroni's correction of *P*-value was evaluated when multiple comparison analysis was used.

## Results

A total of 462 NPC cases and 511 controls were recruited for the present study. All subjects were ethnic Cantonese. The age ranges of the NPC patients (45.9 ± 12.2 years) and the control subjects (45.7 ± 15.6 years) did not differ (P = 0.22, Chi-square test) and no significant differences between cases and controls in the frequency matched variable age were observed at the time of the interview. Of these recruited subjects, 417 NPC cases and 495 controls were eligible for testing at the 194Arg/Trp site, whereas 425 NPC cases and 501 controls were eligible for testing at the 399Arg/Gln site. We did not observe a higher male/female ratio in NPC cases than in controls. The NPC patients consumed fewer cigarettes (16.1 pack-years) than did controls (22.8 pack-years; P = 0.04, Chi-square test); the median cigarette consumption was 20 pack-years in both groups. Age, gender and smoking status of the NPC cases and the controls are summarized in Table 1.

### XRCC1 polymorphisms and NPC risk

Genotyping (Table 2) showed that the frequencies of the XRCC1 194Arg and 194Trp alleles were 69.0% and 31.0% among controls, respectively, and 76.0% and 24.0% among NPC patients. The distribution of XRCC1 Arg194Trp genotypes among controls (Arg/Arg, 47.5%; Arg/Trp, 43.8%; Trp/Trp, 8.7%) was consistent with values predicted by the Hardy-Weinberg equilibrium (P = 0.85, Chi-square test). The frequencies of these three genotypes among NPC patients (Arg/Arg, 55.7%; Arg/Trp, 39.8%; and Trp/Trp, 4.5%) were significantly different from controls (P = 0.009, Chi-square test). The distribution of the Arg/Arg, Arg/Gln and Gln/Gln genotypes at the Arg399Gln site among controls was 53.9%, 40.1% and 6.0%, which was in accordance with the Hardy-Weinberg equilibrium (P = 0.72, Chi-square test). The distribution of these Arg399Gln genotypes among NPC patients (56.7%, 35.8% and 7.5%, respectively) did not differ from the controls (P > 0.05, Chi-squared test).

For the *XRCC1 *Arg194Trp polymorphism, individuals with the XRCC1 Trp194Trp genotype had a nearly 0.50-fold decreased risk of developing NPC compared to those with the Arg194Arg genotype (OR = 0.48; 95% CI, 0.27–0.86, P = 0.01); the risk for developing NPC for individuals with the Arg/Trp genotype did not appear to differ significantly (OR = 0.79; 95% CI, 0.60–1.05). For the XRCC1 Arg399Gln polymorphism, the risk for developing NPC did not differ between individuals with the Arg/Arg genotype and those with the Arg/Gln genotype (OR = 0.82; 95% CI, 0.62–1.08) or the Gln/Gln genotype (OR = 1.20; 95% CI, 0.69–2.06)(Table 2). Since the gender frequency was not matched between the groups, we further analyzed the association between the *XRCC1 *Arg194Trp and Arg399Gln polymorphisms and the risk of developing NPC by gender. Multivariate regression analyses showed that males carrying the *XRCC1 *194Trp/Trp genotype had a reduced risk of developing NPC (OR = 0.32; 95% CI, 0.14–0.70; *P *= 0.004) compared with those carrying the Arg/Arg genotype; risk in males was not effected by the 194Arg/Trp heterozygous genotype (OR = 0.77; 95% CI, 0.54–1.10). No association between *XRCC1 *Arg194Trp variant genotype and the risk of developing NPC was found in females. The OR values were 0.78 (95% CI, 0.46–1.33) and 1.11 (95% CI, 0.47–2.61), respectively, for the *XRCC1 *194Arg/Trp and 194Trp/Trp genotypes. Moreover, there was also no association between Arg399Gln variant genotype and the risk of developing NPC in either males or females (Table 3).

The association of *XRCC1 *Arg194Trp and Arg399Gln polymorphisms and the risk of developing NPC was then analyzed by smoking status. Among smokers, subjects carrying the *XRCC1 *194Trp/Trp genotype had an OR of 0.34 (95% CI, 0.14–0.82; P = 0.01), which was significantly lower than the OR among nonsmokers carrying the Trp/Trp genotype (OR = 0.77; 95% CI, 0.35–1.70) or the Arg/Trp genotype (OR = 0.73; 95% CI, 0.48–1.11) (*P *< 0.01, test for homogeneity). However, no association between Arg399Gln variant genotype and the risk of developing NPC in either smokers or nonsmokers was observed in this study (Table 4).

We examined the association between *XRCC1 *Arg194Trp and Arg399Gln polymorphisms and the risk of developing NPC by age. Though the subjects more than 60 years old carrying the *XRCC1 *194 Trp/Trp or Arg/Trp genotype had lower ORs of 0.27 (95% CI, 0.03–2.52) and 0.48 (95% CI, 0.23–0.99), respectively; the *P*-value was not significant after Bonferroni's correction. Similar results were also obtained from the analysis of the association between the *XRCC1 *Arg194Trp polymorphism and the risk of developing NPC in subjects less 60 years old. No association between the Arg399Gln variant genotype and the risk of developing NPC was observed in either age group (Table 5).

## Discussion

Humans have developed complex DNA repair systems to defend against the harmful consequences of DNA damage and safeguard the integrity of the genome. The human XRCC1 gene, which encodes a protein that plays a central role in the single-strand break repair or base excision repair pathway [19], was identified by its ability to restore DNA repair and mapped to the long arm of chromosome 19q13 [20]. The gene encodes XRCC1 and three other DNA repair enzymes: DNA ligase I at its 3' end, DNA polymerase β, and polyadenosine diphosphate (ADP)-ribose polymerase (PARP), which are involved in the excision and repair of single-strand breaks and in recombinational repair pathways [21,22].

We observed frequencies of 0.26 for the XRCC1 399Gln allele and 6.0% for the Gln/Gln genotype, which are very similar to other studies on Chinese populations [23,24] but significantly lower than those reported for a Caucasian population (0.335 and 11.5%, respectively, n = 1240; P < 0.05) [25]. Studies of associations between the polymorphisms and cancer risk have been inconsistent, possibly because of linkage disequilibrium with a determinant variant of the XRCC1 gene [26]. A close linkage between the 194Trp and 399Arg alleles was previously reported in the Chinese population [27].

This pilot study analyzed two polymorphisms of the XRCC1 gene in relation to the risk of NPC: the amino acid substitutions Arg to Trp at codon 194 and Arg to Gln at codon 399. Arg194Trp resides in the linker region separating the DNA polymerase β domain from the PARP-interacting domain and Arg399Gln residues in the breast cancer susceptibility gene 1 product carboxyl-terminus domain. Both are likely to affect the function of the XRCC1 protein. Chinese hamster ovary cell lines with mutations in XRCC1 have a reduced ability to repair single-strand DNA breaks and are hypersensitive to ionizing radiation and alkylating agents [28].

To date, there is only one published NPC study reporting the associations between NPC risk and XRCC1 polymorphisms. An OR of 0.64 (95% CI, 0.43–0.96) was observed among individuals with the Arg280His or His280His genotypes, whereas no association was noted between polymorphisms at codon 399 of XRCC1 and disease [29]. In this study, we observed a lower risk of developing NPC in individuals with the XRCC1 codon194 Trp/Trp genotype (OR = 0.48, 95% CI, 0.27–0.86). The protective effect of the codon 194 Trp allele observed in our study is consistent with several studies of cancers and XRCC1 genotype. Ratnasinghe et. al. observed a decreased risk of lung cancer associated with the presence of at least one variant allele (OR = 0.7, 95% CI, 0.4–1.2) among Chinese tin miners [30]. Subjects with a codon 194 Trp allele were also at decreased risk of squamous cell carcinoma of the head and neck (OR = 0.8, 95% CI, 0.5–1.3) [31] and bladder cancer (OR = 0.6, 95% CI, 0.3–1.0)[32]. However, there are apparently conflicting data showing that the Arg194Trp genotype increases the risk of head and neck cancer (OR = 2.61) [33] and breast cancer [17].

Although several phenotype analysis studies have associated the 399Gln allele with high levels of DNA adduct formation and increased frequency of sister chromatid change, we failed to observe any association between the XRCC1 Arg399Gln polymorphism (alone or in combination with smoking) and risk of developing NPC. This result is generally consistent with previous findings for lung cancer [25], bladder cancer [34], esophageal cancer [15], and malignant lymphoma [35]. However, there are again conflicting data in the literature. For example, this polymorphism has previously been shown to be a risk factor for lung cancer [36] and head and neck cancer [31] in smokers, yet it has also been shown to be protective for bladder cancer [32], pancreatic adenocarcinoma [16], and gastric cardia cancer [37]. The conflicting results may stem from the complexity etiology of cancer with regard to exposure to carcinogens, DNA repair genotypes or other genetic factors, and the small sample size.

Cigarette smoke is a major cause of a variety of malignancies including cancers of the larynx, oral cavity and pharynx, esophagus, bladder, and lung. Numerous studies have consistently shown that cigarette smoke may play an important role as an environmental etiological factor in the development of NPC in China [2,38]. Cigarette smoke contains a myriad of genotoxic agents and carcinogens such as nitrosamine 4-(methylnitrosamino)-1-(3-pyridyl)-1-butanone (NNK). XRCC1 399Gln/Gln genotype carriers reportedly have a decreased capacity for repair of NNK-induced sister chromatid exchange [11,39]. Although the biochemical and biological characteristics of these XRCC1 variants have not been fully determined, Lunn et al. [40] reported that the XRCC1 399Gln allele was significantly associated with higher levels of placental aflatoxin B1-DNA adducts and glycophorin A somatic mutations. In this study, we observed a significant protective influence of the XRCC1 194Trp/Trp genotype (OR = 0.34, 95% CI, 0.14–0.82) on NPC risk in smokers, suggesting effect modification by tobacco smoking. The results of the present study are generally consistent with previous findings in a relatively large lung cancer study, which showed a protective effect of the XRCC1 194Trp allele against tobacco-related lung cancers (OR = 0.86; 95% CI, 0.77 – 0.95). They also reported that the XRCC1 399Gln/399Gln genotype was associated with a decreased cancer risk among heavy smokers (OR = 0.71, 95% CI, 0.51–0.99), but an increased cancer risk among light smokers (OR = 1.38, 95% CI, 0.99 – 1.94) [41]. We also observed a significantly lower risk of NPC (OR = 0.32; 95% CI, 0.14–0.70) in males carrying the XRCC1 194Trp/Trp genotype. Laboratory and epidemiologic data have indicated that men and women react differently to tobacco and environmental carcinogens [42,43]. Exactly how cigarette smoking and DNA repair polymorphisms affect the DNA repair capacity is unknown. One possible mechanism is that at high levels of exposure, the DNA repair capacity is saturated even in individuals with a higher repair capacity [44,45]. Our findings may explain such a gender difference. The high incidence of NPC in women cannot be attributed to smoking alone in Chinese and other Asian populations where smoking among women is relatively uncommon. Our results suggest that analysis of germline polymorphisms and epigenetic alterations should be further evaluated in these male populations, particularly in epidemiologic studies of the risks of NPC.

## Conclusion

In summary, our study suggests that the XRCC1 Trp194Trp variant genotype is associated with a reduced risk of developing NPC among Cantonese living in southern China. The decrease in risk of NPC associated with this functional polymorphism was more pronounced among smokers and males. No association was observed between the risk of developing NPC and the XRCC1 Arg399Gln polymorphism alone or jointly with smoking, gender, and age. Because of the limited selection of cases, our findings should be confirmed in a larger study.

## Abbreviations

NPC, nasopharyngeal carcinoma; EBV, Epstein-Barr virus; CI, confidence interval; OR, odds ratio; GSTM1, Glutathione S-transferase M1; XRCC1, X-ray repair cross complementing group 1.

## Competing interests

The author(s) declare that they have no competing interests.

## Authors' contributions

**C**Y, MXP, HMY and DL carried out the molecular genetic studies, drafted the manuscript. HLF and IE participated in the design of the study. ZYX, LDX and SJY conceived of the study, participated in its design and coordination and helped to draft the manuscript. All authors read and approved the final manuscript.

## Pre-publication history

The pre-publication history for this paper can be accessed here:



## Figures and Tables

**Table 1:** Characteristics of age, gender and smoking status of cases and controls

**Variable**	**Cases**(***n *= 462**)	**Controls **(***n *= 511**)
**Gender**		
Male	339 (73.4)^a^	252 (49.1)
Female	123 (26.6)^a^	259 (50.9)
**Age range (yr)**		
<60	395 (85.5)^b^	422 (82.6)
≥60	67 (14.5)^b^	89 (17.4)
Mean age in years (S. D.)	45.9 (12.2)	45.7 (15.6)
**Smoking status**^a^		
No	164 (35.5)^c^	353 (69.1)
Yes	298 (64.5)^c^	158 (30.9)
Pack-years smoked		
<20	103 (34.6)^d^	70 (44.3)
≥20	195 (65.4)^d^	88 (55.7)
Mean pack-years smoked (S.D.)	16.1 (17.2)	22.8 (17.3)
Median pack-years smoked (S.D.)	20 (15.6)	20 (17.3)

^a^*P *= 0.0000, χ^2 ^= 58.85; ^b^*P *= 0.22, χ^2 ^= 1.53; ^c^*P *= 0.0000, χ^2 ^= 109.77; ^d^*P *= 0.04, χ^2 ^= 4.15

**Table 2:** Association between risk of NPC and XRCC1 genotypes among cases and controls

Genotype	Cases No. (%)^a^	Controls No. (%)^b^	Unadjusted OR	Adjusted OR^c ^(95%CI)	*P *value
***XRCC1 *Arg194Trp**					
Arg/Arg	232(55.7)	235 (47.5)	**1.00**	**1.00**	
Arg/Trp	166(39.8)	217(43.8)	0.80	0.79(0.60–1.05)	0.10
Trp/Trp	19(4.5)	43(8.7)	0.48	**0.48**(0.27–0.86)	0.01*
Allele of Trp	0.24	0.31			
***XRCC1 *Arg399Gln **					
Arg/Arg	241(56.7)	270(53.9)	**1.00**	**1.00**	
Arg/Gln	152(35.8)	201(40.1)	0.81	0.82(0.62–1.08)	0.16
Gln/Gln	32(7.5)	30(6.0)	1.20	1.20(0.69–2.06)	0.53
Allele of Gln	0.25	0.26			

^a ^417 cases and 495 controls were eligible tested in condon 194 genotype variant.

^b ^425 cases and 501 controls were eligible tested in condon 399 genotype variant.

^c ^Adjusted OR were calculated with the XRCC1 194Arg/Arg or 399Arg/Arg genotype as the reference group and adjusted for age, sex, smoking status.

*P-value remained significant after Bonferroni's correction.

**Table 3:** Association between *XRCC1 *condon 194 and 399 polymorphisms and NPC risk stratified by gender

Genotype	Male				Female			
	
	Cases	Controls	Adjusted OR^a ^(95% CI)	*P *value	Cases	Controls	Adjusted OR^a ^(95% CI)	*P *value
***XRCC1 *Arg194Trp**								
Arg/Arg	175	120	**1.00**		57	115	**1.00**	
Arg/Trp	121	106	0.77(0.54–1.10)	0.16	45	111	0.78(0.46–1.33)	0.36
Trp/Trp	10	21	**0.32(0.14–0.70)**	0.004*	9	22	1.11(0.47–2.61)	0.82
***XRCC1 *Arg399Gln **								
Arg/Arg	177	130	**1.00**		64	140	**1.00**	
Arg/Gln	114	105	0.81(0.57–1.15)	0.24	38	95	0.82(0.47–1.43)	0.48
Gln/Gln	22	15	1.04(0.52–2.11)	0.91	10	15	1.28(0.49–3.36)	0.62

^a^Adjust OR were calculated with the XRCC1 194Arg/Arg or 399Arg/Arg genotype as the reference group and adjusted for age, smoking.

* *P*-value remained significant after Bonferroni's correction.

**Table 4:** Association between *XRCC1 *condon 194 and 399 polymorphisms and NPC risk stratified by smoking status

Genotype	Non smoking				Smoking			
	
	Cases	Controls	Adjusted OR^a ^(95%CI)	*P *value	Cases	Controls	Adjusted OR^a ^(95%CI)	*P *value
***XRCC1 *Arg194Trp**								
Arg/Arg	78	157	**1.00**		154	78	**1.00**	
Arg/Trp	58	155	0.73(0.48–1.11)	0.14	108	62	0.87(0.57–1.33)	0.52
Trp/Trp	10	29	0.77(0.35–1.70)	0.52	9	14	**0.34**(0.14–0.82)	0.017*
***XRCC1 *Arg399Gln **								
Arg/Arg	85	185	**1.00**		156	85	**1.00**	
Arg/Gln	50	140	0.73(0.47–1.12)	0.14	102	60	0.90(0.59–1.38)	0.62
Gln/Gln	11	18	1.42(0.63–3.23)	0.40	21	12	0.91(0.41–1.99)	0.81

^a^Adjusted OR were calculated with the XRCC1 194Arg/Arg or 399Arg/Arg genotype as the reference group and adjusted for age, sex.

* *P*-value remained significant after Bonferroni's correction.

**Table 5:** Association between *XRCC1 *condon 194 and 399 polymorphisms and NPC risk stratified by age range (yr)

Genotype	<60				≥60			
	
	Cases	Controls	Adjusted OR^a ^(95% CI)	*P *value	Cases	Controls	Adjusted OR^a ^(95%CI)	*P *value
***XRCC1 *Arg194Trp**								
Arg/Arg	189	190	**1.00**		42	45	**1.00**	
Arg/Trp	151	179	0.85(0.62–1.16)	0.30	17	38	0.48(0.23–0.99)	0.05*
Trp/Trp	17	37	0.52(0.28–0.96)	0.04	1	6	0.27(0.03–2.52)	0.25
***XRCC1 *Arg399Gln **								
Arg/Arg	210	228	**1.00**		27	42	**1.00**	
Arg/Gln	131	159	0.83(0.61–1.13)	0.23	25	41	0.84(0.38–1.86)	0.67
Gln/Gln	22	25	1.09(0.58–2.05)	0.79	10	6	1.75(0.48–6.44)	0.40

^a^Adjust OR were calculated with the XRCC1 194Arg/Arg or 399Arg/Arg genotype as the reference group and adjusted for sex, smoking status.

*P-value was not significant after Bonferroni's correction.
